# The usage of Mate Select, a web-based selection tool for pedigree dogs for promoting sustainable breeding

**DOI:** 10.1186/s40575-020-00094-8

**Published:** 2020-10-19

**Authors:** Mateja Janes, Thomas W. Lewis, Joanna J. Ilska, John A. Woolliams

**Affiliations:** 1 The Roslin Institute, Royal (Dick) School of Veterinary Studies, University of Edinburgh, Easter Bush, Midlothian, EH25 9 RG Scotland, UK; 2 The Kennel Club, Clarges Street, London, W1J 8AB UK; 3 Schoool of Veterinary Medicine and Science, University of Nottingham, Sutton Bonington, Leicestershire, LE12 5RD UK

**Keywords:** Mate Select, Inbreeding, Breeds, Diversity, Management, Breeding, Tools, Dog, Usage

## Abstract

**Background:**

Inbreeding is a phenomenon that accumulates through the mating of relatives within closed populations, such as pedigree dog breeds, and results in reduced genetic variation within breeds, and may lead to poorer health and fertility from inbreeding depression. The impact of inbreeding is driven by the selection and mating of parents, but information on choices to reduce inbreeding is difficult to assess for individual breeders. Tools to inform dog breeders on the current state of the inbreeding and the relationships among possible parents are potentially useful for providing guidance towards choices that are more beneficial to the breed. However, their utility depends on their usage and this study examines the usage of Mate Select, a web-based tool offered by The Kennel Club, covering 222 breeds for a period of 7 years following its launch in 2011.

**Results:**

The average usage was 2830 searches/week in 2012 with a slight fall of 2.2% per year (P < 0.001) to 2480 searches/week in 2018. Of these, 4% originated from outside the UK, across all continents except Antarctica, with the majority coming from English speaking countries. Searches/week showed a cyclical pattern with two cycles of 26.0 and 50.1 weeks. Since Mate Select’s launch there has been a steady increase in searches from mobile devices, from 11% in 2012 to 43% in 2018. For the 197 breeds with at least 10 dams registered with the Kennel Club during the study period, there was a relationship between usage and registrations, with the average number of searches as a multiple of the number of dams increasing from 2 to 10 for breeds with up to 70 dams and declining towards 2 again for the largest breeds with approximately 20,000 registered dams. However, there remained substantial variation among breeds of similar size, and breeds for which EBVs had become available during the study period had a 2.46 fold greater frequency of searches per registered bitch (P < 0.001), but this was not linked directly to the publication of EBVs.

**Conclusions:**

Mate Select has sustained and substantial usage, although there is also substantial variation in usage among breeds, which offers an opportunity to develop further guidance.

## Plain English summary

Inbreeding is the mating of related individuals that have one or more ancestors in common. In earlier eras, the repeated mating of close relatives was a common practice in dogs and livestock when forming breeds. However, the accumulation of inbreeding is a serious issue in pedigree dogs because it reduces the genetic variation within breeds, may increase the prevalence of inherited disorders, and more generally may have detrimental effects on fertility and viability. One breeder alone cannot control a breed’s rate of inbreeding as it is determined by the breeding decisions for the entire group of contemporary dogs in that breed. Therefore, managing problems from inbreeding is a demanding social problem for breeders and their breed and kennel clubs. Tools can assist breeders by informing them on the current state of inbreeding in their breeds.

Mate Select is one such tool, a free online resource developed by The Kennel Club (KC) in conjunction with the Animal Health Trust. It supports breeding decisions for 222 pedigree breeds recognised by the KC by providing some basic information about any dog or bitch registered with the KC, and any putative mating. This information includes results of health tests, Estimated Breeding Values (EBVs) for complex traits such as hip and elbow dysplasia where these are available, the inbreeding coefficients of the parents and proposed offspring. However, the impact of providing this information will depend on how well the tool is used, which this study examined.

The results show Mate Select has substantial usage, including some international usage mainly from English speaking countries, with users increasingly seeking access via mobile rather than static devices (predicted to be the majority by the time of publication of this paper). Numerically larger breeds had more searches made, with an average (over all breeds) of 4.1 searches per registered dam. The greatest rate of searching per registered dam was for breeds with approximately 70 unique registered dams over the period studied, which spanned 7 years. There was large variation between breeds of the same numerical/census size, and those breeds that had EBVs introduced during the study period had more searches made.

## Background

Inbreeding is a phenomenon present in all species that is the result of mating related individuals. Historically, this has been a common breeding practice in companion animals [1] and in livestock, to increase the predictability of breeding, and this process has underpinned the formation of pedigree breeds. Inbreeding inevitably accumulates within closed populations, such as pedigree dog breeds, and its accumulation reduces the genetic variation within the breed. Inbreeding promotes homozygosity, and consequently can increase the risk from the effects of deleterious recessives [2]. This accumulation is measured by the increasing level of relationships among pairs of individuals in the population, and is usually summarised by the average coancestry (or kinship) within the current breeding population, and the inbreeding coefficients of individuals. These measures are typically calculated from the recorded pedigree and have values between 0 and 1, where 0 is defined by a base generation chosen from the pedigree (often the recorded founders) to act as a reference point, and where 1 represents a completely inbred individual where every locus is expected to be homozygous. Numerous studies have been conducted using these inbreeding coefficients to characterise inbreeding and population structure within and between pedigree canine breeds, e.g. [3]. While these coefficients are informative, it is managing the rate of inbreeding, which is a measure of the rate at which the relationships within the breed accumulate, that is most important for maintaining a genetically robust breed – the lower the better.

The definitions and concepts on rates of inbreeding were developed by Wright [4] and it is well established that small census sizes of breeds promote higher rates of inbreeding, as does selection [5] e.g. for conformation or against lethal recessives. The science for managing the rate of inbreeding over multiple generations of selection in any population is well-established using optimal contribution algorithms [6, 7] and is implemented in practical breeding schemes (e.g. in livestock breeding, [8]). The challenge with these procedures is that they require management of the selection and entire breeding structure. One breeder cannot control the rate of inbreeding in isolation as it is influenced by all those that participate in breeding the next generation, and is determined by the aggregate impact of their individual breeding practices. Therefore, managing problems from inbreeding is a social problem for the breeders and their breed and kennel clubs. The task is challenging, as the best possible outcomes require highly coordinated actions among the many small-scale breeders of pedigree dogs [9]. To facilitate these actions, tools are required to inform a breeder on the current state of inbreeding in their breed and the status of individuals within the breed.

One such tool is Mate Select, which was developed by The Kennel Club (KC), and is a free online resource which was developed in conjunction with the Animal Health Trust (https://www.thekennelclub.org.uk/news/2015/april/mate-select-an-online-tool-for-your-dog-breeders/). Mate Select supports pedigree dog breeders in making breeding decisions by providing some basic information about any dog registered with the KC. The information provided includes DNA tests for deleterious mutations, and phenotypic results of health screening (e.g. eye tests, hip scores) and Estimated Breeding Values (EBVs) for complex traits such as screening evaluation of hip and elbow dysplasia [10] where these are available, and the inbreeding coefficient for any dog in the pedigree or the offspring from a proposed mating. Consequently, breeders can avoid the spread of lethal recessives, select to improve other complex traits, and avoid matings that are closer than would be advisable. Mate Select assists in implementing some of the measures that can be used to restrict inbreeding rates within dog breeds described by Windig and Lewis [11] e.g. restricting kinship of parents, and excluding animals with high inbreeding coefficients.

The impact of any tool will depend strongly on its usage, yet the authors are unaware of other publications describing usage of such tools. Therefore, this study examines the overall usage of Mate Select over 7 years following its release in 2011, and focuses on requests for information on kinship in proposed matings. This usage is further explored in terms of the geographic spread, the mode of access, and to what degree the usage of Mate Select usage is related to breed popularity, availability of EBVs and recorded health tests are summarised.

## Results

The results examine the use made by breeders of the ‘kinship’ information provided by Mate Select, which can be obtained for any putative mating between a registered dog and a registered bitch. This gives information on the inbreeding coefficient of the bitch and the dog and the offspring that would be produced from such a mating, which may not ultimately take place. In what follows, a request for information is defined as a search, and a distinction is made between a bitch, which is a registered female, and a dam, which is a registered female with registered offspring.

### Usage of Mate Select over time

**Fig. 1:**
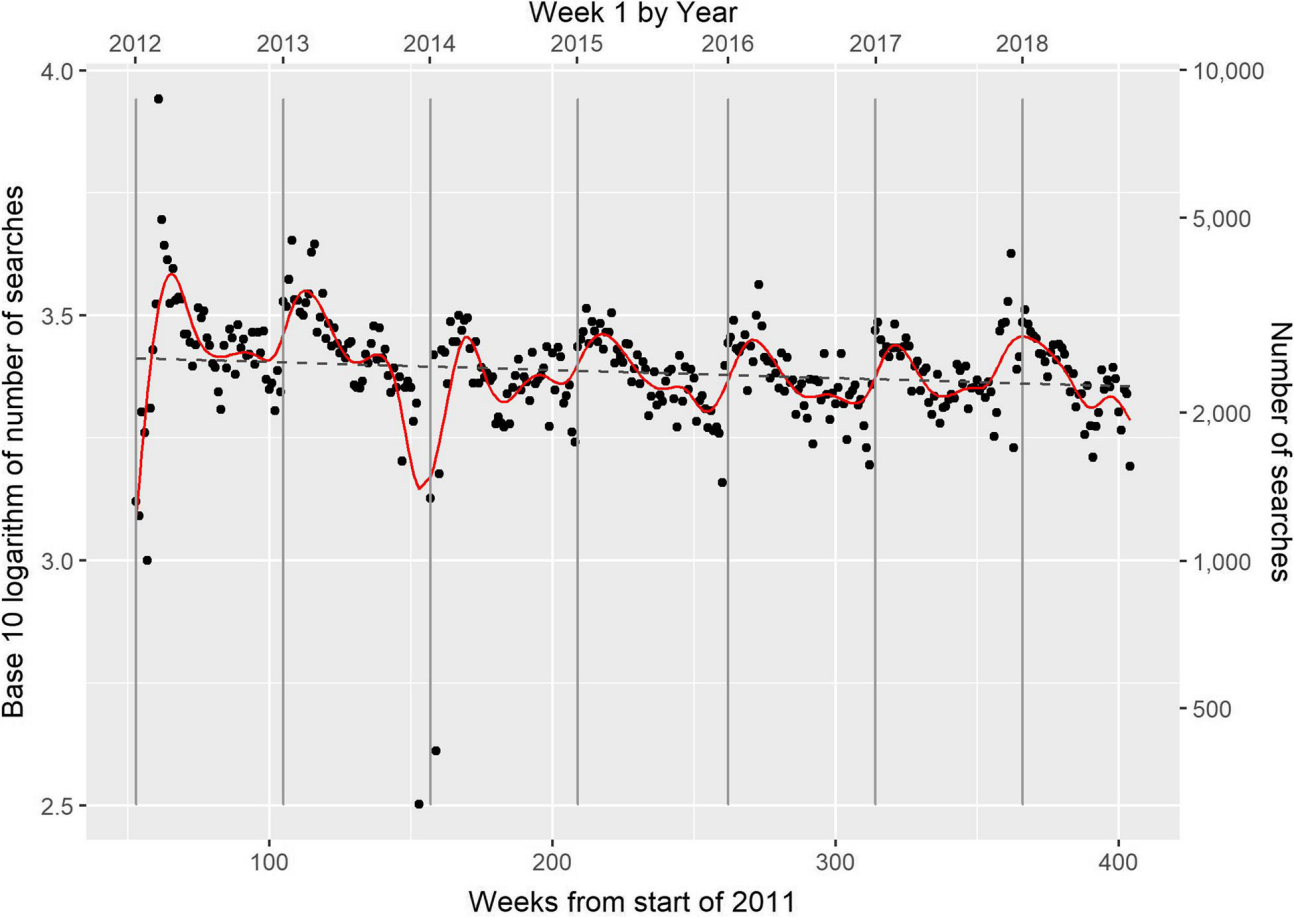
The number of searches per week over the study period. The base 10 logarithm of the number of searches plotted against weeks since the start of 2011. The period shown is from the first week of 2012 onwards, with the vertical grey bars denoting the first week of each year from 2012 to 2018 inclusive. The red line shows the trend estimated from mixed-model smoothing using cubic splines, and the dashed grey line is the estimated underlying linear trend

The average number of searches per week during the first full calendar year (2012) was 2831/week, which had fallen by 12.4% to 2480 per week in 2017, which was the last calendar year with complete data. There was a substantial peak at its initial release in May 2011 followed by a marked drop towards the end of the 2011. After this time, the pattern of usage (displayed in Fig. 1) became more stable. Figure 1 shows a marked cyclicity, which was confirmed by the pattern which shows approximate periods of 50.1 weeks for the major cycle and 26.0 weeks for the minor, and the periodogram obtained from the Fourier decomposition is shown and described in Supplementary Information 1. The periodicity corresponds to a major period of activity in spring and, less clearly, in autumn, with less activity in summer and midwinter. The slope of the estimated time trend on the log10 scale for the period shown in Fig. 1 was − 1.64 × 10− 4 (s.e. 0.45 × 10− 4) per week, which corresponds to a fractional drop in usage of 1.9% per annum. The amplitude in the annual pattern of search volume has decreased since the launch. Over 2015 to 2017 inclusive, the busiest 5 weeks in the year had 1.8 fold more searches than the slowest 5 weeks.

### Usage by location

**Fig. 2:**
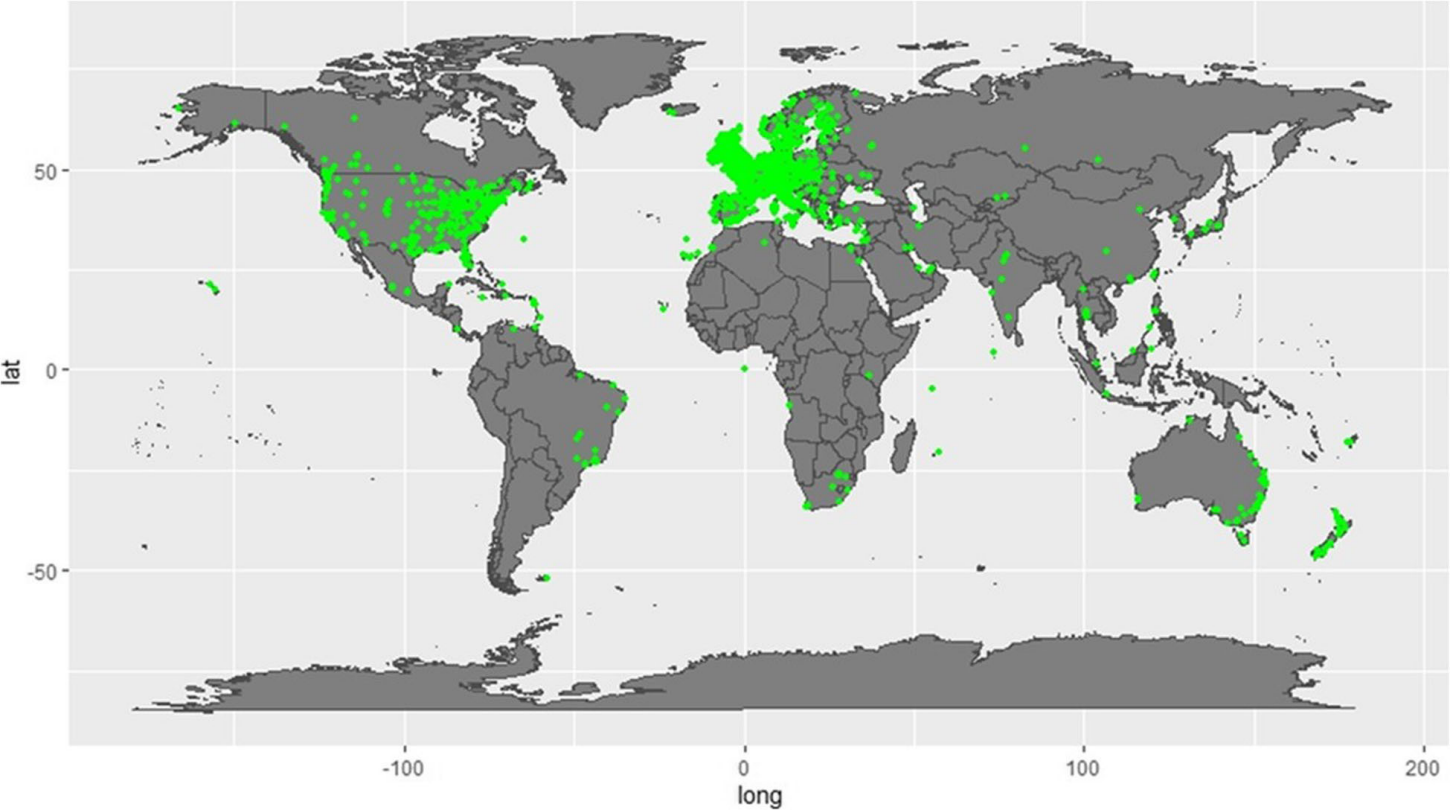
World map showing the origin of searches. Green dots represent locations of unique IP addresses from which searches originated

Figure 2 presents the worldwide map of searches, and illustrates that Mate Select has been accessed from all continents except for Antarctica. Table 1 shows the calculated frequency of searches per continent and countries from which that majority of the searches were made, and indicates that the primary usage outside the UK was from English speaking countries, specifically Ireland and United States.

**Table 1:** The frequency of searches from different continents and selected countries. Frequency of searches across continents and the consitutent countries with more than a fraction of 0.001 of all searches. Public IP addresses and addresses returned as “NA” by the R package “IPtoCountry” are excluded. The fraction presented is of the total number of searches included

Continents and countries	Frequency of searches	Fraction of searches
United Kingdom	569,175	0.956
Europe (without UK)	19,755	0.033
Ireland	4716	0.008
Netherlands	3364	0.006
Finland	2379	0.004
Italy	1347	0.002
North America	4492	0.008
United States	3541	0.006
Oceania	684	0.001
Asia	1020	0.002
Africa	255	0.000
South America	218	0.000
TOTAL	595,599	

### Searches on mobile and static devices

**Fig. 3:**
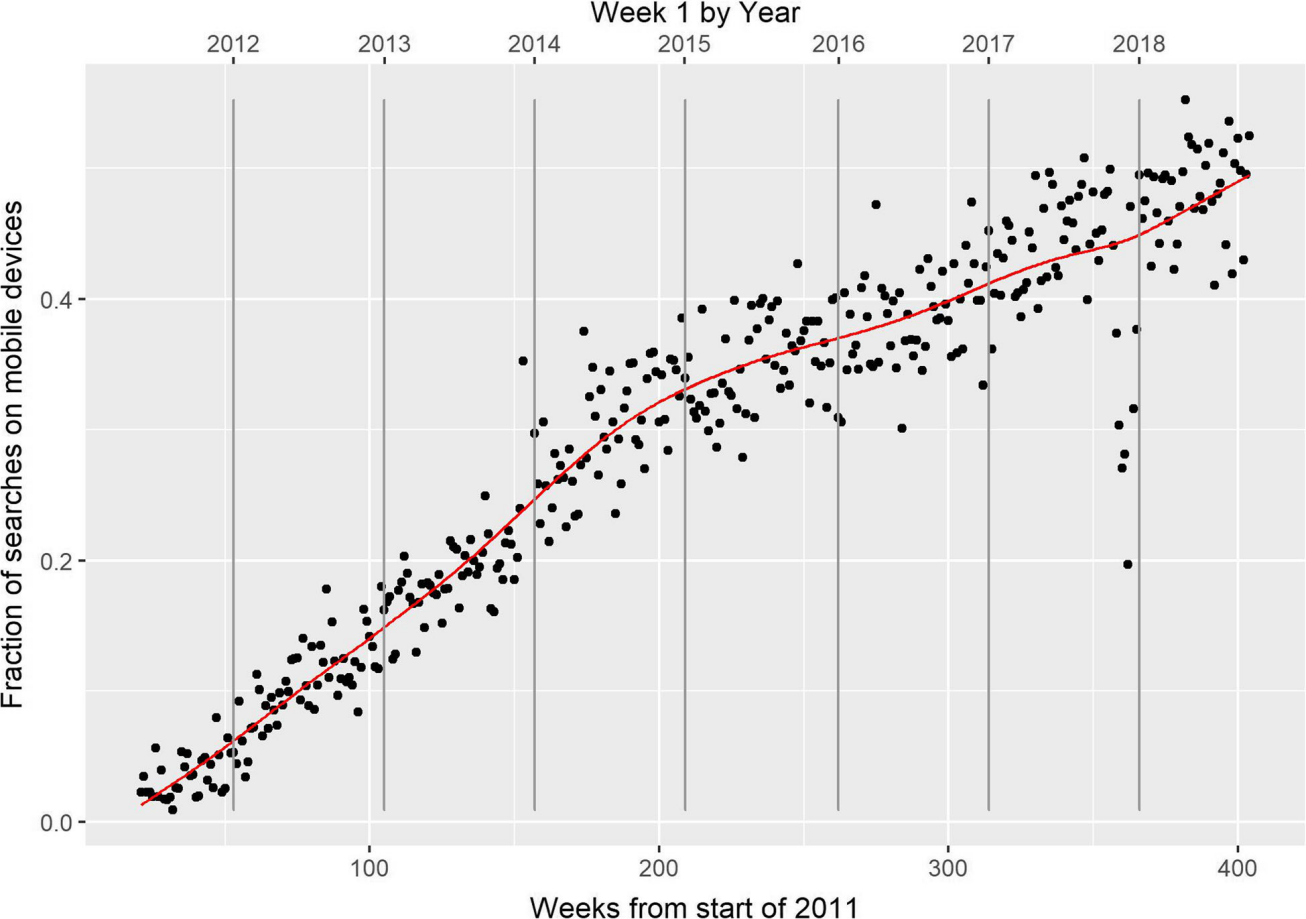
The fraction of searches on mobile devices over time. The change in the fraction of searches using mobile browser agents over time. The red line is the smoothed line obtained from mixed-model smoothing using cubic splines. The vertical grey lines denote the initial weeks of 2012 to 2018 inclusive

Figure 3 shows the fraction of all searches that were conducted through browser agents on mobile devices. While at the launch of Mate Select searches from static devices constituted the vast majority, the fraction of searches from mobile devices has increased rapidly and steadily. In the first full calendar year, mobile searches were only 11% of total searches, but this increased to 43% in 2017. This fraction was still increasing at a rate of 5% p.a. from 2016, and had reached 50% over the last 6 weeks of the studied data.

### Breed variation in the usage of Mate Select

**Fig. 4:**
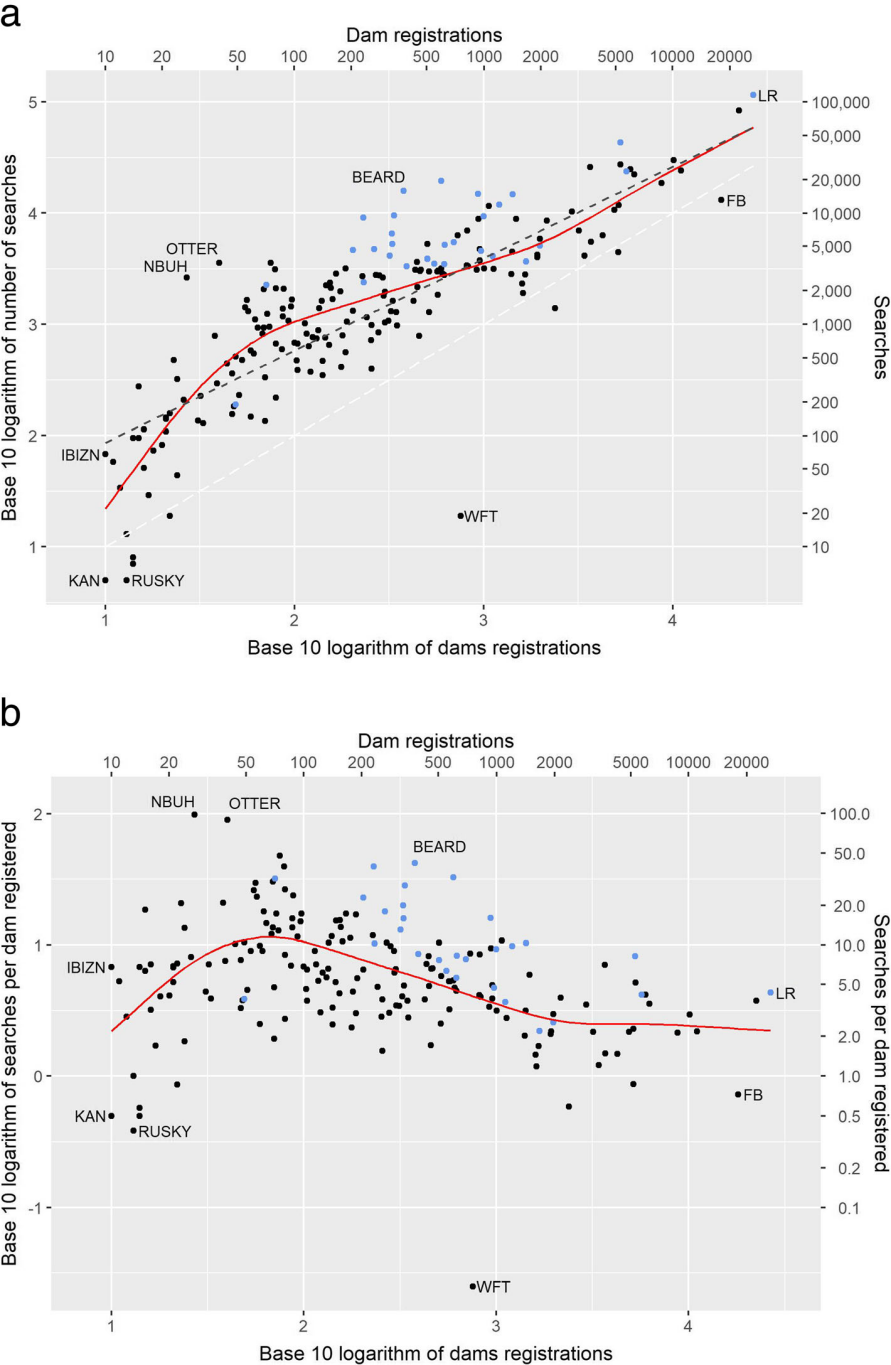
The relationship between frequency of searches per breed and dams registered. **a** A graph showing the base 10 logarithm of the number of searches per breed plotted against the base 10 logarithm of the number of registered dams per breed. The red line shows the trend obtained from the mixed-model smoothing spline fitted with Eq. (2), and the dashed line is the underlying linear trend of y = 1.109 + 0.827x. The white dashed line is y = x. The points shown in light blue are the EBV-track breeds, which had EBVs provided by The Kennel Club by the end of the study period. The labelled breeds from the convex hull and are: LR, Labrador Retriever; FB, French Bulldog; WFT, Wire Fox Terrier; RUSKY, Russian Toy; KAN, Turkish Kangal Dog; IBIZN, Ibizan Hound; NBUH, Norwegian Buhund; OTTER, Otterhound and BEARD, Bearded Collie. **b** A transformation of (**a**) where the y axis is now the number of searches as a multiple of the number of registered dams plotted against the log of the number of registered dams per breed. The red line shows the trend obtained from the mixed-model smoothing spline fitted with Eq. (2). The points shown in light blue are the EBV-track breeds, which had EBVs provided by The Kennel Club by the end of the study period. Labelled breeds are the same as in (**a**)

Figure 4a shows the relationship between Mate Select searches per breed and the unique number of dams registered for the 197 breeds with at least 10 dams registered with the Kennel Club in the study period. It shows a clear positive relationship between usage and breed registrations, with a more rapid increase in searches in relation to registrations for breeds with smaller census size up to ~ 50 dams, followed by a lower rate on the logarithmic scale thereafter. The slope of the overall linear trend on the logarithmic scales is < 1, (0.827, s.e. 0.042) which indicates that the numbers of searches per registered bitch is declining as the census size increases. Note that the searches made on Mate Select include those for bitches that do not ultimately have registered offspring.

The relationship between census size measured by the number of dams, and the frequency of searches as a multiple of the number of dams is shown more explicitly in Fig. 4b, which is derived from the model and data presented in Fig. 4a. The predicted number of searches increases from 2.2 searches per dam for breeds with census size of 10 dams, to 10.5 searches per dam with census size of 100 dams, then decreases to 3.5 and 2.4 searches per dam for census sizes of 1000 and 10,000 dams respectively. The peak of the trend line in Fig. 4b is 11.5 searches per dam for breeds with census size of 70 dams. For the extreme census size of the Labrador Retriever the search rate was 4.32 searches per dam.

The Labrador Retriever is also one of 29 EBV-track breeds which are defined here as breeds that had EBVs published for either hip dysplasia or elbow dysplasia, or both, by the end of the study period. As shown by Fig. 4a, these breeds attracted more searches per registered dam, among breeds of the same size. The estimate from the analysis was that the EBV-track breeds had 2.46 fold more searches than other breeds of the same census size (*P* < 0.001). This prompted an additional analysis described in Supplementary Information 2 which showed this difference was not associated with publication *per se* and existed prior to publication of EBVs. The scale of the variation about the smoothed trend obtained from the analysis shows that, when adjusted for census size, a breed at the upper quartile of searches would have 1.82 fold more searches than a breed at the lower quartile, more than expected from simple Poisson variation.

The 9 breeds forming the convex hull of the distribution shown in Fig. 4a and b are listed in Table 2. These breeds form the ‘edge’ of the joint distribution of breed size and searches per breed on the logarithmic scale. For example, Labrador Retriever (LR) and Ibizan Hound (IBIZN) had a higher number of searches per dam given their census size whereas the Turkish Kangal Dog (KAN), Wire Fox Terrier (WFT) and French Bulldog (FB) are extreme in having a relatively low number of searches given their size, with WFT being particularly extreme having 40 times more registered dams than searches. In contrast, the Norwegian Buhund (NBUH) had 98 times more searches than registered dams.

**Table 2:** The breeds forming the convex hull of the distribution of search frequency and breed size. Tag – breed abbreviation used in Fig. 4a; Registered Dams – census size of the breed, measured by the number of unique registered dams over the period of study; Searches in Mate Select – total searches for the breed; Searches/Bitch – the average number of searches per registered dam; EBV-Track – availability of EBVs by the end of study period

Breed	Tag	Registered Dams	Searches in Mate Select	Searches/Dam	EBV-Track
Otterhound	OTTER	40	3572	89.30	No
Norwegian Buhund	NBUH	27	2650	98.15	No
Ibizan Hound	IBIZN	10	68	6.80	No
Turkish Kangal Dog	KAN	10	5	0.50	No
Russian Toy	RUSKY	13	5	0.38	No
Wire Fox Terrier	WFT	757	19	0.03	No
French Bulldog	FB	18,119	13,105	0.72	No
Labrador Retriever	LR	26,723	115,451	4.32	Yes
Bearded Collie	BEARD	378	15,907	42.08	Yes

## Discussion

Over the last decades, pedigree dog breeding has attracted public scrutiny over a number of welfare issues associated with conformation (e.g. [12–14]), such as syringomyelia in Cavalier King Charles Spaniels and some other toy breeds [15] or Brachycephalic Obstructive Airway Syndrome in French Bulldogs and other brachycephalic breeds [16], and the management of deleterious recessive conditions segregating in many breeds [1, 17–19]. These issues have a complex background, with the practices which originally led to creation of breeds, i.e. inbreeding and breeding to a defined standard for physical and temperament characteristics, nowadays becoming a hindrance to the genetic health of the populations [16].

The problems in management of the gene pool in pedigree dog populations are easier to identify than to solve for two reasons: firstly, the population structure of most breeds is vastly different from typical pyramid structure observed in many livestock species, with many breeders contributing their animals to the breeding pool, and any individual breeder having at best only a weak control of the overall breeding population. Secondly, pedigree breeds are closed populations by definition, with many pedigree breeds having very small genetic bases. Web tools such as The Kennel Club’s Mate Select offer a means of sharing technical information on the genetic state of a breed and so can promote informed decision-making among all breeders within a breed towards beneficial outcomes. However, such outcomes rely on the tools being used and, to the authors’ knowledge, this study is the first to examine to what degree this is so. Mate Select is an important subject for such a study as it is one of few tools managed by a national kennel club, consolidating the complete records of pedigree dogs registered in the country, for decades. The tool offers information on 222 breeds with a wide range of census sizes, and collectively these breeds have over 230,000 registrations per year. The results show that the tool has a large community of users going beyond the UK, which has remained relatively constant in number since its launch, but usage can vary dramatically for breeds of similar census size, and is greater for those breeds with EBVs available.

The measures of usage and breed population size used in this study approximate the breeding population of a breed. Usage was assessed by the number of searches querying putative mating pairs as this represents the key decision point when the future gene pool is being shaped and the planning of matings is fundamental to the pedigree breeder. Not all of the bitches that were the subject of searches were bred from, i.e. not all became dams, however their presence in the search data represents one part of the breeder’s decision-making process. The measure of breed size was the numbers of bitches that produce registered offspring, i.e. are dams for the breed, as this most closely represents the size of breeding resources available for a breed. This choice avoids biases arising from variation in litter size, which would have influenced the total number of registrations for a breed, and complications associated with the mating ratios of dams to sires. With these definitions, the simple average across all breeds was 4.1 searches per dam over the period of the data and indicates significant use of Mate Select.

Ideally, for genetic management one might wish for more searches per dam for the smaller breeds as choices are more critical when the genetic base is narrow, but this was not observed for the breeds with the smallest census sizes studied here. However, there are reasons why this expectation may be unreasonable. Firstly, when a breed has only a few dams there are also few dogs available, so the mate choices are limited by census size too. Secondly the dams of those numerically small breeds may be owned by very few breeders and it is feasible that the information stored in Mate Select may also be maintained privately. Moreover, the UK ‘population’ may actually only be an outpost/subset of a larger (but still small) global breed population, which may have maintained pedigree databases independent of national registries. Beyond this group of the numerically smallest breeds, the expectation of more searches per dam for smaller breeds was evident, with a peak at 70 registered dams (see Fig. 4b) followed by a decline as breed populations became larger. However, the variation around the trend line shown in Fig. 4a shows there is considerable variation attributable to breed culture or structure among breeds of similar size. Among the extreme deviations, the reasons for the profoundly low search frequency for the Wire Fox Terrier remains a mystery, while the high search frequency for Norwegian Buhund has coincided with the release of a DNA test for cerebellar ataxia affecting this breed [20]. While the last decades have seen a multitude of DNA tests developed for various breeds, no other coincidences of extreme search frequencies with emerging DNA tests were observed.

Some of the structural and cultural causes of variation among breed in searches per dam, after accounting for size, may prove to be predictable as demonstrated by the results for the EBV-track breeds in this study. These breeds have participated in BVA/Kennel Club recording schemes long enough to accumulate sufficient data for EBVs for hip and/or elbow dysplasia to be produced by the end of the study period. Participation in such schemes is itself a step towards collectively addressing a breed’s health problems, and some have consequently shown demonstrable improvement in the hip scores [21], and researching the phenotypic scores would encourage usage of Mate Select. EBVs first became available for some UK breeds in March 2014, midway through the study period, and the number of breeds and the scope of the EBVs for particular breeds has expanded at different times since then. When EBVs are produced for a breed, the estimates of genetic merit are more accurate and encompass the whole of the breed, not just those that have been phenotyped. Whilst the hypothesis that publication of the EBVs may be responsible for the increased searching on Mate Select for these breeds is plausible, the analysis in Supplementary Information 2 provides no support for this and shows that differences in usage existed prior to publication. The study has highlighted a number of factors that may be considered for increasing usage as tools are developed, beyond the importance of the providing good genetic content, such as EBVs, as a means of maintaining and promoting usage. Firstly, the rapid growth in demand for accessing the tools on mobile devices was very clear, and the trends shown in Fig. 2 would predict that the majority of users would now be using Mate Select from mobile devices. This requires (i) the underlying software to be flexible enough to cope with the demand for providing information on different platforms, and (ii) as importantly, how the information is presented needs to account for this variation.Secondly, the tools can have global usage, although for Mate Select usage from outside the UK was relatively small (< 5%) and primarily from within Europe and English-speaking countries. This too raises questions on presentation, not only in relation to language and terminology, but also on content. For example, EBVs currently provided for hip and elbow dysplasia within Mate Select are based on the scoring system developed by British Veterinary Association, UK. Thus, the EBVs are not directly comparable to EBVs developed in other countries and based on other scoring systems (e.g. Orthopedic Foundation for Animals in breeds registered with American KC, or Federation Cynologique Internationale in most other countries) [22]. While combining the scores from different systems to calculate EBVs may not be feasible at this time, as it would require merging of worldwide pedigree databases, a useful first step would be to generate and present conversion formulae, which would be more meaningful to international users. Thirdly, usage has a clear cyclicity with periods of comparatively heavy use within the year. Awareness of this should be accounted for in the planning of down-times (e.g. for maintenance) and the launch of improvements to avoid discouraging usage in both the short- and long-term.

## Conclusions

In conclusion, this novel study has demonstrated that the Mate Select tool is well-used, although there is substantial variation in usage among breeds. The high degree of usage among breeders offers opportunities for improving the guidance given to pedigree dog breeders towards more sustainable management of their gene pools. This will depend on the accessibility, design and content of the tool but achieving this wider objective will depend on the decision-making of the breeder: in particular, whether or not the choice made after having searched alternative mating options is the most beneficial for the health of the breed.

## Methods

### Data

The data was extracted from two large databases. The first was the anonymised log of searches using Mate Select, recorded by KC since its inception in May 2011, to the end of September 2018. In this context, a search is a request for information on a proposed mating of two specified individuals. Over the period, there were 946,632 searches, covering 213 breeds. For each search, the information available included the date of search, the mode of access, and regional location of the user. The second database was that for pedigree, which consisted of 10,182,582 individuals from 224 breeds. As well as the recorded pedigree, this database contained information on sex, birth and registration dates, elbow and hip scores (where available) and phenotypes such as coat colour. A total of 1,857,384 dogs were registered over the period of the Mate Select data used. These two databases were linked through KC’s unique dog identifier, which was recorded for each search and included in the pedigree data. Some 29 breeds had been long-standing participants in the BVA/Kennel Club recording scheme for hip and elbow dysplasia (https://www.thekennelclub.org.uk/services/public/mateselect/ebv/Default.aspx), and the recorded phenotypes were supplemented by EBVs for one or both dysplasia published over the course of the study period, but with varying starting dates; these are referred to as ‘EBV-track’ breeds.

### Data analysis

Various aspects of the Mate Select database were used to characterise the extent and mode of usage over time. These aspects included: (i) the overall rate of access to Mate Select and its changes over time since its inception; (ii) the geographical spread of users as assessed by the available information on IP addresses; (iii) the usage by mobile or static devices assessed from information on the web browser agent; (iv) the evidence for differences in usage according to breed and how this was linked to breed popularity, and the availability of EBVs.

### Search volume

The search data was aggregated into the total number of searches per day, and then per week, where weeks started on Mondays. The data covered a period of 384 weeks in total. The weekly counts were then transformed using base 10 logarithms and a linear model was fitted incorporating a simple linear time trend plus a smoothing spline over time. The model fitted was

1 $$ \boldsymbol{y}=\mathbf{1}m+\mathbf{t}b+f\left(\mathbf{t}\right)+\mathbf{e} $$

where **y** is the logarithm of the count, *m* is an intercept with **1** a vector of 1’s, **t** is the vector of week numbers, *b* the linear regression coefficient on time, *f*(**t**) is the cubic smoothing spline over time, and **e** is the vector of residuals. The model was fitted using ASReml-R [23] which estimates the optimum smoothing parameter following Verbyla et al. [24]) and White et al. [25]. An initial scan of the outcome showed an initial burst of usage, before a more steady state was reached, and evidence of cyclicity. Therefore, the data from 2011 were removed, and the model refitted to the remaining data. The Lomb-Scargle periodogram was estimated [26] to examine the cyclicity after removing the linear trend estimated by the model above, and was fitted in the R-package ‘spectral’.

### Geographical spread

The geographical origins of searches were extracted from the IP addresses using the R-package ‘IPtoCountry’ [27] which identified the country of origin of the IP address, the city and/or region within the country, zip code, latitude and longitude. All the 946,632 searches originated from 126,613 unique IP addresses, of which some were public IP addresses or were of unknown location and were therefore deleted. After their removal, 126,609 unique IP addresses remained representing 595,599 (63%) of all searches. The latitudes and longitudes were used to plot the searches on the world map using R package ‘rworldmap’ after adjustment to the base map using packages ‘ggmap’ and ‘mapplots’.

### Computer or mobile access

Information on the mode of access was available from the browser agent used in the search. A simple approach was taken to partition searches between those from a static device (e.g. PC, Apple Mac), or a mobile device by searching for ‘mobile’ in the text of the identifier browser agent included in the Mate Select database. In this search, tablets were included as a mobile. The fraction of mobile searches was then calculated and analysed with the same model as Eq. 1.

### Searches in relation to breed

To assess the usage in relation to breed popularity the number of searches per breed in Mate Select was compared to the breed popularity as assessed from the database on pedigree registrations. The Mate Select searches were quantified as the total number of searches per breed over the period of study. The measure of breed popularity was the number of active dams, defined as those appearing in the registration of offspring over the period covered by the data on Mate Select.The number of dams was therefore extracted for each breed and compared to the number of searches per breed over the same period. The breeds with less than 10 active dams over the period of the data were not included in the analysis. The subset of EBV-track breeds was also considered as a classifier among breeds. Information on health tests for single loci was considered ubiquitous to all breeds and its use in the selection of mates is beyond the scope of this present study.

A mixed linear model similar to Eq. (1) was fitted

2 $$ \mathbf{y}=\mathbf{1}m+\mathbf{x}b+f\left(\mathbf{x}\right)+\mathbf{z}c+\mathbf{e} $$

but in (2): **y** is the base 10 logarithm of total searches; **x** is the base 10 logarithm of the number of dams registered; and the additional term accounted for whether or not the breed had EBVs published by The Kennel Club at any point in the study period, with **z** a simple 0/1 indicator variable and *c* its effect. Since the model is on a logarithmic scale, Eq. (2) can be transformed into a model for the ratio of searches to registered dams, by subtracting **x** from both sides.

## Supplementary information

**Additional file 1.** The spectral analysis of the weekly usage in Mate Select.

**Additional file 2.** The impact of EBV publication on search frequency.

## Data Availability

The databases from which data was extracted for analysis are owned by The Kennel Club and are not available without approval from The Kennel Club. The summary data extracted and analysed in this study was anonymised, but contains indirect information on locations. This summary data will be given a persistent DOI and archived in University of Edinburgh DataVault and access will be given on reasonable request to the authors.
