# Supplementary material for: The usage of Mate Select, a web-based selection tool for pedigree dogs for promoting sustainable breeding
Source: Canine Med Genet. 2020 Oct 19;7:14. doi: 10.1186/s40575-020-00094-8 (PMC7574414; doi:10.1186/s40575-020-00094-8)

**Supplementary Information 1**

**The spectral analysis of the weekly usage in Mate Select.**

As stated in the Materials & Methods a spectral analysis was carried out on the weekly usage date shown in Figure 1 after having removed the linear trend obtained by fitting Eqn.[1], i.e. $\mathbf{y}-\mathbf{1}\hat{m}-\mathbf{t}\hat{b}$. The Lomb-Scargle periodogram (Zechmeister and Kurster, 2009) was obtained directly from using the R-package ‘spectral’ after carrying out the Fourier decomposition. The periodogram is shown in Figure S1 with all data and the frequencies of maximum amplitude shown are 0.0199 and 0.0384 corresponding to major and minor cycles with periods of 50.1 and 26.0 weeks respectively. Removing the two outliers at the end of 2012 and start of 2013 (see Figure 1 in main text) increased the normalised PSD of the major cycle and increased the false alarm probability (FAP) of the spectrum for frequencies other than the major cycle at 50.1 weeks, although the FAP of the minor cycle at 26.0 weeks remained <0.05.

**Figure S1. The Lomb-Scargle periodogram for the weekly search frequency.** The input data was the natural logarithm of numbers of searches from Week 1 in 2012 to Week 39 in 2018 after removing the linear time trend. The black line is the normalised power spectral density (PSD), and the dashed red line is the false alarm probability (FAP). The normalised PSD is the squared amplitude of the cycle divided by twice the variance of the sample, and the FAP is a Type 1 error defined as the probability that a white noise signal with no periodic component would produce a peak of the observed magnitude or larger.


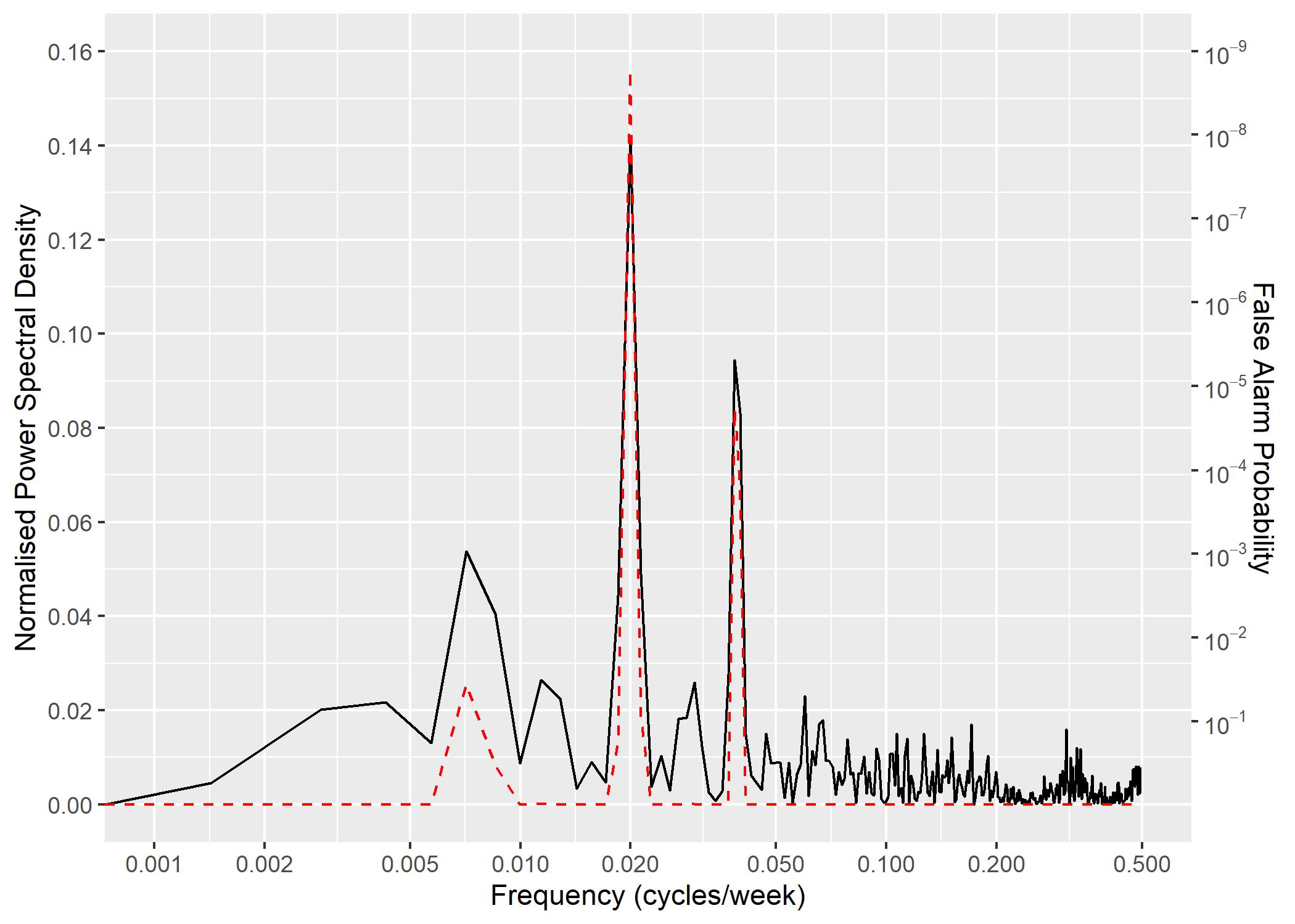

Supplement: Supplementary file 1 — Additional file 1. The spectral analysis of the weekly usage in Mate Select. [file 40575_2020_94_MOESM1_ESM.docx]
