# Supplementary material for: The usage of Mate Select, a web-based selection tool for pedigree dogs for promoting sustainable breeding
Source: Canine Med Genet. 2020 Oct 19;7:14. doi: 10.1186/s40575-020-00094-8 (PMC7574414; doi:10.1186/s40575-020-00094-8)

**Supplementary Information 2**

**The impact of EBV publication on search frequency**

The results in the main paper show that EBV-track breeds had 2.46-fold more searches than a breed with a similar number of registered dams that was not EBV track. At the start of the period of study there were no EBVs but during the study period EBVs appeared for 29 breeds in three tranches. To test the impact of publication of an EBV *per se* on search frequency the first tranche of 15 breeds were investigated, which had had EBVs launched in March 2014. The search frequency prior to launch in the 52 weeks from Week 50 in 2012 to Week 49 in 2013 inclusive (Period 1) was compared to the frequency post launch in the 52 weeks from Week 50 in 2014 to Week 49 in 2015 (Period 2). The timings of these periods were informed by the results in the main paper which showed an approximately annual cycle with lowest search frequency at approximately Week 50, and so the impact of the truncation points was minimised. The periods also avoided searches associated with publicity at the time of launch and in the subsequent breeding season of 2014. To account for time trends the search frequency in these breeds was compared to 15 breeds without EBVs and chosen either (i) by the number of active registered dams from week 50 in 2012 to week 49 in 2015 inclusive (Case A) or (ii) by the number of searches made during Period 1 (Case B). No other selection criteria were used.

**Case A**. The data consisted of 60 observations on search frequency for 15 pairs of breeds in Periods 1 and 2. The analysis focused on two hypothesis and were tested by paired t-tests with 14 d.f. on the natural logarithm of the counts. The first of these was whether there was evidence that the EBV-track breed had a greater search frequency than the control; the second was evidence of an interaction between periods and EBV-track/control breed, in particular whether the difference between EBV-track breeds and control breeds increased from Period 1 to Period 2. EBV-track breeds had 2.46-fold greater searches in Period 1 (P<0.001), which was consistent with the outcome of the analysis in the main study where all breeds with 10 or more dams were included. In Period 2, the differential between EBV-track and control breeds was less, not greater, with only 2.08-fold greater searches; this difference in search frequency between EBV-track and control breeds remained statistically significant (P<0.001), but there was no evidence of an interaction between the breeds and periods (P>0.05). In summary the differences between the EBV-track breeds and controls existed prior to the publication of EBV’s and there was no evidence that this difference was affected by publication.

**Case B**. The pre-existing difference in search frequency between the paired breeds prompted a second examination with a different pairing of breeds based on search frequency in Period 1. As with Case A the data consisted of 60 observations on search frequency for 15 pairs of breeds in Periods 1 and 2. The analysis took the form of linear regression of the logarithm of the number of searches in Period 2 on the logarithm of the number of searches in Period 1, with the hypothesis that EBV-track breeds would differ in intercept or slope or both. Figure S2.1 shows the relationship between Period 2 and Period 1 observations for both EBV-track breeds and control breeds. There was no evidence of a difference between these groups (P>0.05).

**Figure S2.1. The impact of EBV publication on search frequency.** The number of searches in Period 2 plotted against the number of searches in Period 1 plotted on a base 10 logarithmic scale for 15 breeds with EBVs published in March 2014 (cornflower markers) and 15 control breeds (black markers) paired on the number of searches in Period 1. The grey dashed line represents the line *y*=*x*, and the solid red line is the linear regression for all points *y*=0.019+0.963*x*.


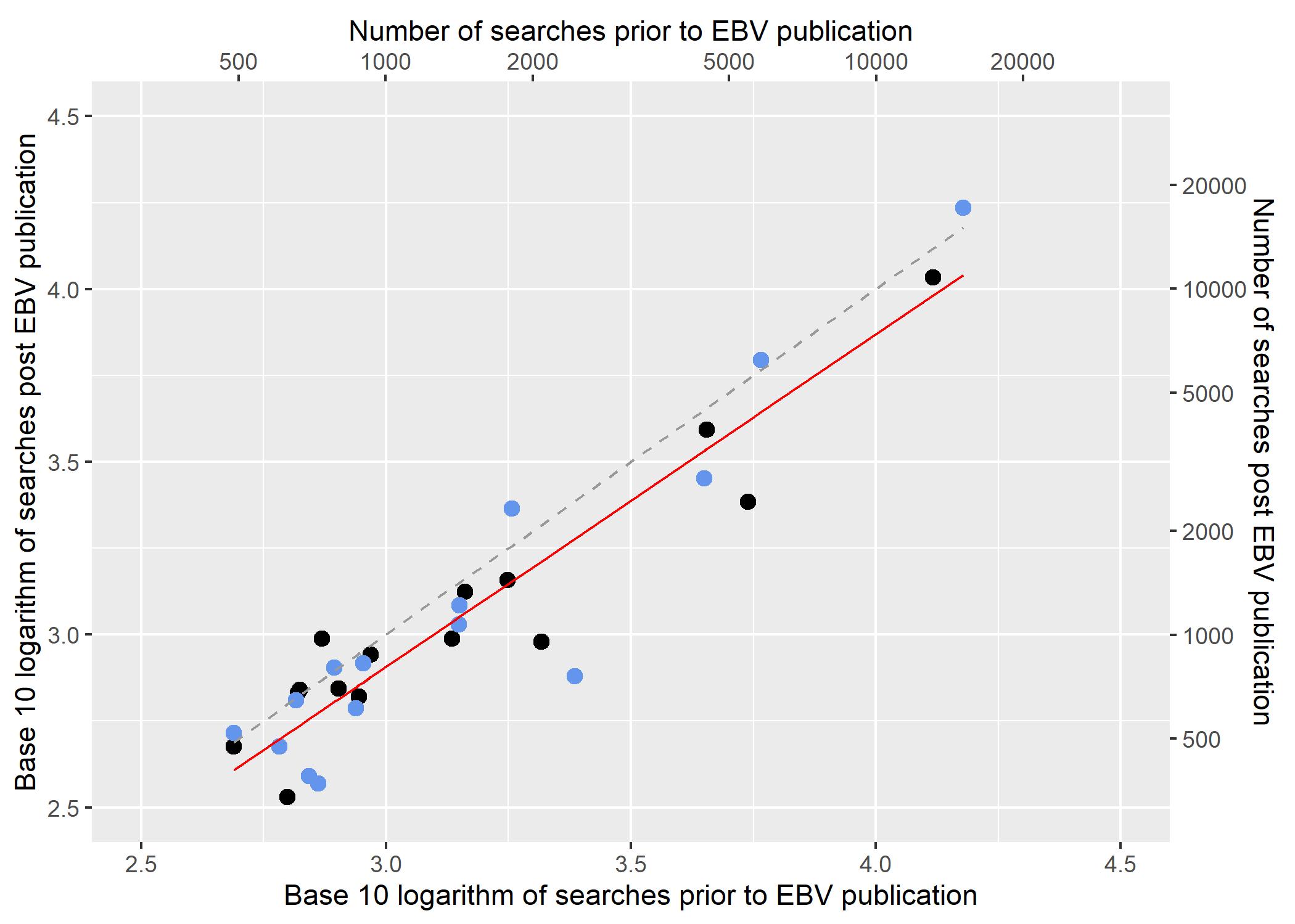

Supplement: Supplementary file 2 — Additional file 2. The impact of EBV publication on search frequency. [file 40575_2020_94_MOESM2_ESM.docx]
